# Anephrogenic phenotype induced by *SALL1* gene knockout in pigs

**DOI:** 10.1038/s41598-019-44387-w

**Published:** 2019-05-29

**Authors:** Masahito Watanabe, Kazuaki Nakano, Ayuko Uchikura, Hitomi Matsunari, Sayaka Yashima, Kazuhiro Umeyama, Shuko Takayanagi, Tetsushi Sakuma, Takashi Yamamoto, Sumiyo Morita, Takuro Horii, Izuho Hatada, Ryuichi Nishinakamura, Hiromitsu Nakauchi, Hiroshi Nagashima

**Affiliations:** 1 Meiji University International Institute for Bio-Resource Research, Kawasaki, 214-8571 Japan; 2 Laboratory of Developmental Engineering, Meiji University, Kawasaki, 214-8571 Japan; 3 Department of Mathematical and Life Sciences, Graduate School of Science, Hiroshima University, Hiroshima, 739-8526 Japan; 4 Laboratory of Genome Science, Biosignal Genome Resource Center, Institute for Molecular and Cellular Regulation, Gunma University, Gunma, 371-8512 Japan; 5 Department of Kidney Development, Institute of Molecular Embryology and Genetics, Kumamoto University, Kumamoto, 860-0811 Japan; 6 Division of Stem Cell Therapy, The Institute of Medical Science, The University of Tokyo, Tokyo, 108-8639 Japan; 7 Department of Genetics, Institute for Stem Cell Biology and Regenerative Medicine, Stanford University School of Medicine, Stanford, CA 94305 USA

**Keywords:** Genetic engineering, Stem-cell research

## Abstract

To combat organ shortage in transplantation medicine, a novel strategy has been proposed to generate human organs from exogenous pluripotent stem cells utilizing the developmental mechanisms of pig embryos/foetuses. Genetically modified pigs missing specific organs are key elements in this strategy. In this study, we demonstrate the feasibility of using a genome-editing approach to generate anephrogenic foetuses in a genetically engineered pig model. *SALL1* knockout (KO) was successfully induced by injecting genome-editing molecules into the cytoplasm of pig zygotes, which generated the anephrogenic phenotype. Extinguished SALL1 expression and marked dysgenesis of nephron structures were observed in the rudimentary kidney tissue of *SALL1*-KO foetuses. Biallelic KO mutations of the target gene induced nephrogenic defects; however, biallelic mutations involving small in-frame deletions did not induce the anephrogenic phenotype. Through production of F1 progeny from mutant founder pigs, we identified mutations that could reliably induce the anephrogenic phenotype and hence established a line of fertile *SALL1*-mutant pigs. Our study lays important technical groundwork for the realization of human kidney regeneration through the use of an empty developmental niche in pig foetuses.

## Introduction

Critical shortage of donor organs is a serious challenge in the field of organ transplantation that severely limits opportunities to rescue patients with end-stage organ failure. In recent years, there have been efforts to solve this problem through the generation of functional human tissues and organs using pluripotent stem cells such as induced pluripotent stem (iPS) cells^1–3^. However, generating transplantable solid organs *in vitro* is considered impractical owing to the complex three-dimensional structure and function of human organs^4,5^. Under such circumstances, the novel concept of growing human organs *in vivo* by utilizing the developmental mechanisms of porcine embryos genetically engineered to be unable to form a specific organ has attracted considerable attention [for review, see^6,7^]. The basic approach underlying this concept, considering the pancreas as an example, is to allow a pancreas to develop from exogenous human pluripotent stem cells in growing porcine embryos that lack endogenous pancreatogenesis^8^. One of the key technical elements in the application of this strategy for the generation of various human organs is the creation of genetically modified pigs in which normal organ formation is inhibited.

The existence of master regulator genes responsible for organ and tissue formation has previously been reported in mammals^9–12^. Previous studies in rodents have demonstrated that knockout (KO) of such genes gives rise to phenotypes lacking specific target organs. For example, knocking out *Pdx1*, *Sall1*, and *Foxn1* can induce phenotypes associated with hypoplasia or complete loss of the pancreas, kidneys, and thymus, respectively^13–15^. In addition, Kobayashi *et al*.^16^ and Usui *et al*.^17^ successfully produced xenogeneic pancreases in *Pdx1* KO mice and allogeneic kidneys in *Sall1* KO mice, respectively, using a blastocyst complementation approach. These studies have paved the way for research on human organogenesis that exploits empty developmental niches, the dedicated *in vivo* environments that are left vacant when the normal development of endogenous organs is disrupted, in animals.

The genetic engineering strategies used to induce organogenesis-disabled phenotypes in rodents have not been verified in pigs, except with regard to the apancreatic phenotype^8,18,19^. The aim of this study was to determine whether knocking out *SALL1*, which is known to play a pivotal role in rodent nephrogenesis, could induce the anephrogenic phenotype in pigs. We report here the results obtained after introducing *SALL1* mutations in pigs, including the mutation efficiencies after cytoplasmic injection of transcription activator-like effector nucleases (TALENs) and CRISPR/Cas9 in zygotes, the effects of the mutations on kidney formation in foetuses and progeny, and our success in establishing a line of *SALL1*-mutant pigs characterized by an anephrogenic phenotype.

## Results

### Efficiency of mutation induction by cytoplasmic injection of *SALL1*-targeted Platinum TALEN and CRISPR/Cas9 into parthenogenetic embryos

**Figure 1:**
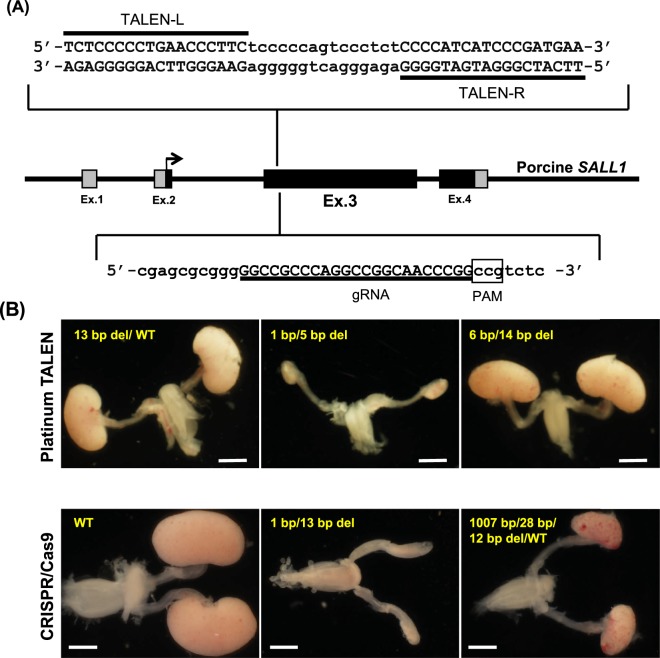
Generation of porcine *SALL1* gene knockout foetuses by genome editing. (**A**) Design of Platinum TALENs and CRISPR/Cas9 targeting the porcine *SALL1* gene, which consists of 4 exons, similar to that of humans. The coding and untranslated regions are indicated by black and grey boxes, respectively. A pair of Platinum TALENs (top) and CRISPR/Cas9 (bottom) target the beginning of exon 3 in porcine *SALL1*. The protospacer adjacent motif (PAM) is a short specific sequence following the target DNA sequence that is essential for cleavage by Cas9 nuclease and is indicated with a white box. **(B)** Kidney phenotypes of the genome-edited foetuses with mutant SALL1. Foetuses developed from zygotes injected with Platinum TALENs (upper 3 panels) and CRISPR/Cas9 (lower 3 panels) were examined for nephrogenesis on days 36–37 and 39–40 of gestation, respectively. Kidneys with homozygous frameshift mutations (upper and lower middle) exhibited an anephrogenic phenotype. Foetuses harbouring WT *SALL1* (13 bp del/WT, upper left) or *SALL1* with a small in-frame mutation (6 bp/14 bp del, upper right) developed normal kidneys like those of a WT foetus (lower left). The kidneys of a foetus with complex mosaic mutations (1007 bp/28 bp/12 bp del/WT, lower right) showed mild hypoplasia. Scale bars: 2 mm.

The mutation induction efficiencies of two genome-editing tools, Platinum TALEN^20^ and CRISPR/Cas9, designed to target exon 3 of porcine *SALL1* (Fig. 1A) were investigated following cytoplasmic injection into porcine parthenogenetic embryos at the pronuclear stage. Mutations were induced in over 80% of blastocysts obtained after injection of 2 or 5 ng/μl Platinum TALEN-encoding mRNA (Table 1). However, the blastocyst formation rate was significantly lower after the 5 ng/μl injection than after the 2 ng/µl injection (17.9% vs. 55.8%, P < 0.05), indicating a detrimental effect of TALEN-encoding mRNA with higher concentration.

**Table 1:** Incidence of mutations in porcine embryos after cytoplasmic injection of Platinum TALEN mRNA targeting the *SALL1* gene.

Platinum TALEN mRNA (ng/μl)	Embryos injected*	Embryos cleaved (%)	Embryos developed to blastocysts (%)	Avg. number of cells in blastocysts (mean ± SEM)	Blastocysts analysed	Blastocysts with mutations (%)	Blastocysts with biallelic mutations (%)
Not injected	62	50 (80.6)^a^	36 (58.1)^a^	89.0 ± 8.6^a^	NA	NA	NA
0	94	79 (84.0)^a^	59 (62.8)^a^	79.1 ± 7.4^a^	NA	NA	NA
2	95	71 (74.7)^a^	53 (55.8)^a^	77.2 ± 8.0^a^	24	21 (87.5)^a^	12** (50.0)^a^
5	95	74 (77.9)^a^	17 (17.9)^b^	67.6 ± 16.5^a^	7	6 (85.7)^a^	6 (85.7)^a^

^ab^Values with different superscripts in the same column differ significantly (P < 0.05).

*Parthenogenetic embryos at the pronuclear stage.

**Includes 3 embryos with 3 types of mutations.

NA: not analysed.

Mutation efficiencies were also compared between Cas9-mRNA-based and Cas9-protein (ribonucleoprotein; RNP)-based CRISPR/Cas9 systems. Blastocyst formation rates and mutation rates were compared for various concentrations of guide RNA (gRNA; 2 to 10 ng/µl) and Cas9 (2 to 20 ng/µl). Blastocyst formation rates were stable (49.4–63.2%) and mutations were consistently detected, except at the lowest concentration (2 ng/µl) of RNP tested (62.0–97.0%; Supplementary Table S1).

These results demonstrated that cytoplasmic injection of the two genome-editing molecules, Platinum TALEN and CRISPR/Cas9, into pronuclear-stage embryos could induce mutations in porcine *SALL1* with high efficiency. Subsequent experiments generating mutant foetuses used either 2 ng/µl Platinum TALEN, which achieved a better blastocyst formation rate than the higher concentration, or 20 ng/µl Cas9-RNP, the highest Cas9-RNP concentration and the concentration that achieved the best mutation rate.

### Anephrogenic phenotype of *SALL1*-KO foetuses

*SALL1*-KO foetuses were generated to test whether *SALL1* KO could reliably induce deficiency of nephrogenesis in a porcine model. Platinum TALEN and CRISPR/Cas9 molecules were cytoplasmically injected into *in vitro*-fertilized embryos at the pronuclear stage.

**Figure 2:**
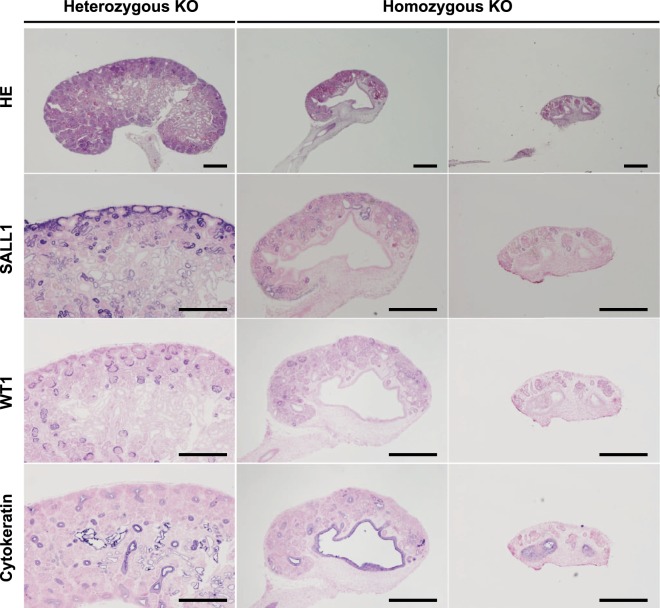
Hypoplastic kidneys of *SALL1*-KO founder foetuses. Foetuses developed from zygotes injected with Platinum TALENs were obtained at day 36–37 of gestation. Left panels: Kidney tissue of a *SALL1* heterozygous founder foetus (M231-8; WT/13 bp del). Middle and right panels: Kidney tissue of two homozygous *SALL1*-KO founder foetuses (M230-1 and M231-6; 5 bp/13 bp del and 1 bp/5 bp del). The homozygous KO foetuses showed severe renal hypoplasia. The signals for SALL1 and WT1 were markedly reduced in the rudimentary kidney tissue of the foetuses with homozygous KO mutations. Scale bars: 500 μm.

Platinum TALEN-injected embryos were transplanted into two recipients, yielding 13 viable foetuses after 36–37 days of gestation (Table 2). A wide variety of *SALL1* mutations were observed in all foetuses (Supplementary Table S2), and a nephrogenic defect or severe renal hypoplasia was observed in nine (69.2%; Table 2 and Fig. 1B). Genomic DNA analysis revealed homozygous frameshift mutations or large deletions in these individuals (Supplementary Table S2). The tissue of the rudimentary kidneys lacking nephron structures mainly consisted of interstitial cells. Immunohistochemical staining revealed slight yet detectable signals of SALL1 and WT1 (Fig. 2). Together, these results suggested the occurrence of mosaic mutations in founder genome-edited foetuses, indicating that *SALL1* gene products, including truncated isoforms, might have been produced in the renal tissue. Rare populations of wild-type (WT) or in-frame mutant cells in the renal tissue could possibly have been left undetected in genomic analysis of the foetal tissues.

**Table 2:** Generation of *SALL1*-KO founder foetuses by cytoplasmic injection of Platinum TALENs and CRISPR/Cas9 into porcine zygotes.

Genome-editing tool (conc.)	Recipient no.	Embryos injected*	Embryos transferred	Embryonic stage at transfer	Pregnancy	Foetuses obtained	Foetuses with mutations (%)	Renal hypoplasia** (%)
Platinum TALENs (2 ng/µl)	M230	179	90	2- to 8-cell	+	8	8 (100)	6 (75.0)
	M231	162	93	2- to 8-cell	+	5	5 (100)	3 (60.0)
	Total	341	183		2	13	13 (100)	9 (69.2)
gRNA/Cas9 (5/20 ng/µl)	#1705	242	157	1-cell	+	9	5 (55.6)	1 (20.0)
	#1706	211	62	2- to 8-cell	+	3	2 (66.7)	2 (66.7)
	Total	453	219		2	12	7 (58.3)	3 (42.9)

^*^IVM/IVF-derived embryos at the pronuclear stage.

^**^Foetuses with homozygous loss-of-function mutations.

In contrast, the remaining four individuals in which nephrogenic defects were not induced had either heterozygous or homozygous mutations involving small in-frame deletions (Fig. 1B and Supplementary Table S2). Truncated SALL1s translated from DNA with only small in-frame mutations seemed to have activity comparable to that of the WT protein.

CRISPR/Cas9-injected embryos were transferred to two recipients, yielding 12 viable foetuses after 39–40 days of gestation (Table 2). Various *SALL1* mutations were detected in seven foetuses (58.3%; Supplementary Table S3), and the anephrogenic phenotype was expressed in three (42.9%; Fig. 1B). Mild renal hypoplasia was observed in one mosaic individual (#1705-5) possessing various mutant and WT cells.

Taken together, the above data indicated that *SALL1* KO could induce an anephrogenic phenotype or severe renal hypoplasia in pigs, just as it does in mice^14^.

Additionally, we determined whether any off-target mutation had occurred in the *SALL1-*KO pig foetuses obtained using Platinum TALEN. No mutations were detected in any of the three off-target sites with the highest target-sequence similarity with the Platinum TALEN used (Supplementary Table S4).

### Genotypic and phenotypic variations in founder *SALL1*-mutant pigs

After confirming the anephrogenic phenotype in the foetuses, we examined whether *SALL1* KO resulted in viable offspring with nephrogenic defects. Platinum TALEN was exclusively used for these experiments, considering that its yield of mutant *SALL1* foetuses was greater than that of CRISPR/Cas9 in the present study, as described in the previous section. Platinum TALEN-injected embryos were transferred to three recipients, yielding 16 offspring in total (Table 3). Genetic analysis detected mutant *SALL1* sequences in 13 offspring (81.3%; Supplementary Table S5); two of these (M253-3 and M253-5) were biallelic mutants with a small in-frame deletion. These founder *SALL1* mutants developed normally. In addition, two of the *SALL1*^mutant (mut)/WT^ piglets (M243-3 and M253-2) had normally developed kidneys. No viable *SALL1*-KO piglets were born with an anephrogenic or renal hypoplasia phenotype.

**Table 3:** Production of genome-edited founder piglets carrying mutant *SALL1* using Platinum TALENs.

Recipient no.	Embryos injected*	Embryos transferred	Embryonic stage at transfer	Pregnancy	Offspring delivered (stillborn)	Offspring with:		
						Mutations (%)	Monoallelic mutations (%)	Biallelic mutations (%)
M243	304	67	Blastocyst	+	3 (1)	3 (100)	3 (100)	0 (0)
M244	212	56	Blastocyst	+	7 (0)	5 (71.4)	5 (71.4)	0 (0)
M253	275	142	2- to 8-cell	+	6 (0)	5 (83.3)	3 (50.0)	2 (33.3)
Total	791	265	−	3	16 (1)	13 (81.3)	11 (68.8)**	2 (12.5)***

*IVM/IVF-derived zygotes at the pronuclear stage.

**Includes 6 piglets carrying 2 or 3 types of mutation.

***Includes one piglet with 5 types of mutations.

Founder animals engineered via cytoplasmic injection of genome-editing molecules in zygotes are prone to mosaic mutations and are characterized by a number of coexisting populations of mutant cells^21–25^; indeed, over half of the mutant piglets carried mosaic mutations (Supplementary Table S5).

To compare mosaicism rates across different tissues, we explored the variations in *SALL1* mutations in tail, kidney, and gonad/germ cells (ovarian tissue or epididymal sperm) from five mosaic offspring (1 female and 4 males). The mutations detected did not differ greatly across the three tissues; however, two mutations were found only in the tail and not in gonads or kidneys (Supplementary Table S6). This finding implies that the mutations detected in tail samples may not be included in germ cells of the mosaic individuals and may therefore not be able to be transmitted to subsequent generations.

### Genotypic and phenotypic features of F1 progeny carrying *SALL1* mutations

Although we could generate founder *SALL1*-mutant offspring with high efficiency via cytoplasmic injection of *SALL1*-targeted Platinum TALEN into *in vitro*-fertilized pronuclear embryos, not a single *SALL1*^−/−^ piglet was obtained. To determine whether the *SALL1*^−/−^ genotype is prenatally lethal in pigs, we investigated whether viable homozygous *SALL1-*KO offspring could be obtained by crossing two mutant founders carrying frameshift mutations. A total of 11 F1 progeny were delivered by two sows in three litters. All F1 offspring were heterozygous mutants or WT piglets; not a single one had the *SALL1*^−/−^ genotype (Table 4).

**Table 4:** Production of F1 progeny by mating of founder pigs carrying *SALL1* mutations.

Breeding pair (founder)		Pig code (F1)	Sex	Genotype
♂	♀			
M253-4 [WT/5 bp del/1 bp/56 bp ins]	M253-6 [WT/91 bp ins]	W304-1	♂	5 bp del/WT
		W304-2*	♂	1 bp ins/WT
		W304-3*	♂	WT
	M244-1 [WT/13 bp/660 bp del]	W307-1	♂	1 bp ins/WT
		W307-2	♂	660 bp del/WT
		W307-3	♀	1 bp ins/WT
		W307-4	♀	1 bp ins/WT
		W307-5*	♀	1 bp ins/WT
	M253-6** [WT/91 bp ins]	W304(2)-1	♀	1 bp ins/WT
		W304(2)-2	♂	5 bp del/WT
		W304(2)-3	♂	1 bp ins/WT

*Stillborn.

**Delivered in the second litter.

**Figure 3:**
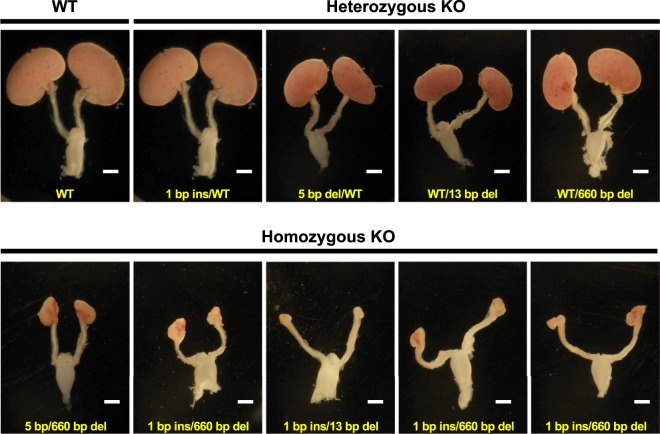
Anephrogenic phenotype in F1 progeny carrying *SALL1* mutations. F1 foetuses on day 40 of gestation were obtained by mating founder *SALL1* mutant pigs. All foetuses with homozygous mutations (lower panels; W307-6, W307-7, W307-8, W307-9, and W307-11) exhibited various phenotypes of renal hypoplasia. Foetuses with heterozygous mutations (upper panels; W307-1, W307-2, W307-4, and W307-10) showed normally formed kidneys like those of a WT foetus (W307-3). Scale bars (white): 2 mm.

**Figure 4:**
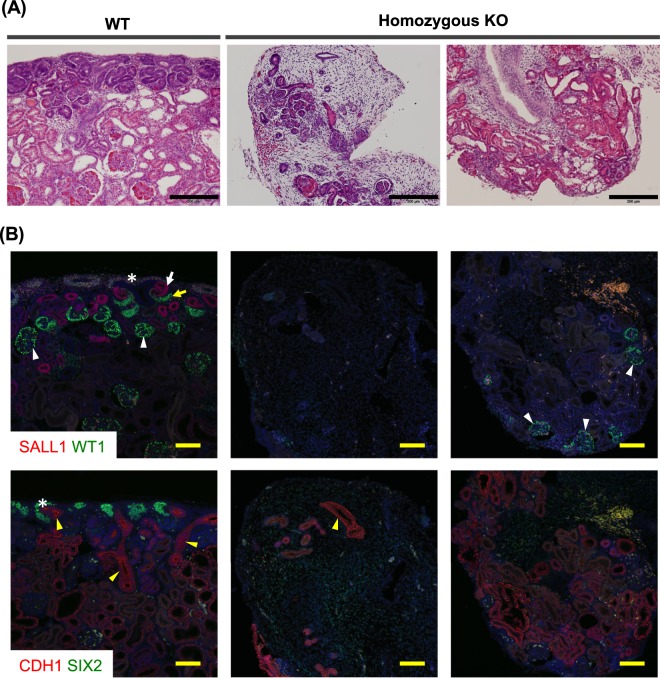
Hypoplastic kidneys of F1 progeny carrying *SALL1* mutations. Left panels: Kidney tissue of a WT foetus (W307-3). Middle and right panels: Kidney tissue of homozygous *SALL1*-KO F1 progeny (W307-8; 1 bp ins/13 bp del and W307-11; 1 bp ins/660 bp del) at day 40 of gestation. (**A**) Histological examination of foetal kidneys by haematoxylin-eosin staining. Homozygous KO foetuses showed kidneys that were disorganized and severely hypoplastic kidneys compared to those of WT foetuses. Black scale bars: 200 µm. **(B)** Immunofluorescence analysis of kidneys of homozygous *SALL1*-KO F1 progeny. Upper panels: SALL1, which was expressed in nephron progenitors (*) and distal nephrons (white arrow) in WT foetuses, was absent in homozygous KO foetuses (middle and right panels). WT1 was expressed in proximal nephrons (yellow arrow) and glomeruli (white arrowheads) in WT foetuses, whereas residual glomeruli (white arrowheads) were detected in KO foetuses. Lower panels: SIX2, which was expressed in nephron progenitors (*) in WT foetuses, was absent in homozygous KO foetuses. Ureteric buds (yellow arrowheads), which expressed CDH1 (E-cadherin), were detected only residually in KO foetuses. Yellow scale bars: 100 µm.

To further investigate the outcome of *SALL1*-KO mutations, we obtained viable foetuses produced by crossing founder *SALL1* mutants (M253-4♂ × M244-1♀, Supplementary Tables S5 and S7) at day 40 of gestation. Twelve F1 foetuses were collected, of which five were homozygous for *SALL1* KO (Supplementary Table S7). These five individuals exhibited an anephrogenic or severe renal hypoplasia phenotype, including disorganized formation of a few glomeruli and tubules (Figs 3 and 4A).

Renal tissue from *SALL1*-KO foetuses and a control WT foetus was subjected to immunohistochemical analysis. In WT tissue, SALL1 was expressed in nephron progenitor cells and distally in immature nephrons, whereas WT1 was expressed in nephron progenitor cells, proximally in immature nephrons, and in glomeruli (Fig. 4B).

In contrast, SALL1 expression was not apparent in the hypoplastic kidney tissue of *SALL1*^−/−^ foetuses (Fig. 4B). SIX2, which is typically expressed in nephron progenitor cells, was absent from the renal tissue of *SALL1*^−/−^ foetuses (Fig. 4B). In addition, formation of nascent renal tubules and ureteric buds, indicated by positive signals for CDH1, was limited (Fig. 4B). Analysis of hypoplastic renal tissue of a *SALL1*^660 bp del/1 bp ins^ foetus (biallelic mutant, 660 bp del/1 bp ins) showed a small number of WT1-positive glomeruli, as well as structures resembling nascent renal tubules or ureteric buds (Fig. 4B). Immunostaining with an antibody that recognizes the C-terminus of SALL1 was performed on rudimentary renal tissue from a different foetus with the same mutation profile (660 bp del/1 bp ins); SALL1 expression could still not be confirmed (Supplementary Fig. S1).

## Discussion

The present study demonstrated that *SALL1*^−/−^ in pigs gave rise to anephrogenic phenotype as shown in mice previously^14^. Creating a pig line exhibiting the anephrogenic trait is a prerequisite for kidney regeneration via the blastocyst complementation strategy.

*Sall1* is an essential gene for kidney formation, and its KO induces nephrogenic defects in mice^14^. However, when a *Sall1* mutation does not lead to loss of function and instead produces a truncated protein, the dominant negative effects of the isoform may cause kidney malformation, as in Townes-Brocks syndrome^26,27^. To induce an anephrogenic phenotype, therefore, a null mutation of this gene is desirable. For this reason, we designed Platinum TALENs and CRISPR/Cas9 with target sites in the upper N-terminal region of SALL1.

We opted to use cytoplasmic injection in zygotes to generate *SALL1-*mutant pigs. While this approach has been used successfully to generate gene KO rodents and pigs^28–33^, it has several limitations, including the production of offspring with undesirable mutations and of mosaic individuals harbouring multiple mutations^21–25^. Indeed, we observed a high incidence of mosaicism in the founder foetuses and offspring obtained in this study. Mosaicism cannot necessarily be detected by DNA analysis of a tissue sample such as a tail snip^34–36^. In fact, some of our founder foetuses that were identified as *SALL1*^−/−^ during tissue DNA analysis, showed faintly positive signs of SALL1 expression in the rudimentary kidney upon immunohistochemistry, thereby suggesting the presence of a small population of cells carrying an undetected mutation or the WT sequence. The prolonged activity of genome-editing molecules after the first cleavage of a zygote is considered to be responsible for the development of mosaicism^37,38^. To inhibit the development of mosaicism, an attempt to advance the time of introduction of genome editing molecules into zygotes^39^ and use of destabilized molecules with shorter reactivity have been reported^40^. A method for the introduction of Cas9 protein instead of mRNA has also been proposed^41,42^, even though our preliminary study did not reveal any discernible effect of inhibiting the development of mosaicism. At present, to our knowledge, there is no reliable measure for preventing the development of mosaicism and further studies are awaited.

Renal hypoplasia and nephrogenic defects varied across the *SALL1*^−/−^ foetuses obtained in the present study. Impaired nephrogenesis in *Sall1*-deficient mice is not uniform in nature; it can present as missing kidneys and ureters or as unilateral or bilateral renal hypoplasia, with each of the three phenotypes occurring at roughly the same frequency^14^. This phenotypic variation could be explained by the involvement of multiple genes besides *Sall1* in kidney development.

Kidneys form through a mutual inductive interaction between the ureteric bud, which emerges from the mesonephric duct, and the metanephric mesenchyme^14^. GDNF (glial-cell derived neurotropic factor) is released from the metanephric mesenchyme^43–45^. GDNF is an important humoural factor that guides the elongation of the ureteric bud into the metanephric mesenchyme. Since SALL1 is a transcription factor that upregulates GDNF, reduction or KO of its expression could downregulate GDNF production, thus impeding proper renal development. GDNF is upregulated by other genes besides *Sall1*, such as *Wt1* and *Pax2*^46–48^. The involvement of affiliated genes in the phenotypic variation in renal hypoplasia following *SALL1* knockout has yet to be determined. Genetic heterogeneity of the non-inbred domestic pig used in this study might be one of the causes of the phenotypic diversity.

Homozygous *Sall1*-KO mice can develop to full term, although the KO is neonatally lethal^14^; however, we failed to obtain any viable *SALL1*^−/−^ piglets among either mutant founders or F1 progeny generated by crossing two mutant founders. Notably, our F1 litter size per sow was apparently smaller than normal. However, *SALL1*^−/−^ foetuses were alive in the uterus at day 40 of gestation. Accordingly, we presume that homozygous *SALL1* KO is prenatally lethal in pigs during the second trimester of pregnancy or later. This supposition is corroborated by our findings from a separate analysis of cloned *SALL1*^−/−^ foetuses produced via somatic cell nuclear transfer (in submission).

Difference in prenatal lethality of *SALL1*-KO mice and pigs may be ascribed to the role of *SALL1* during foetal development in both the species. Further studies are needed to elucidate the influence of the loss-of-function mutation in *SALL1*, which is normally also expressed in non-renal tissues, on the entire embryogenesis process in pigs^49–52^. A relatively immature character of mouse foetuses compared to pig foetuses may also be involved in the survival of *SALL1*-KO mouse to full term.

In conclusion, our current study demonstrates that homozygous *SALL1* KO in pigs induces a nephrogenesis-deficient phenotype in foetuses. We identified mutations that could reliably induce the anephrogenic phenotype and established lines of fertile individuals harbouring these mutations. Our study has laid important technical groundwork for the realization of human kidney regeneration through the use of an empty developmental niche in developing pig foetuses.

## Methods

### Animal care and chemicals

All of the animal experiments including genetic modifications performed in this study were approved by the Institutional Animal Care and Use Committee (IACUC) of Meiji University (IACUC-12-0008, IACUC-17-0004). All recombinant DNA experiments performed in this study were approved by the Gene Recombination Experiment Safety Committee (GRESC) of Meiji University (GRESC-13-7, GRESC-18-5). All experiments were performed in accordance with the relevant guidelines and regulations. All chemicals were purchased from Sigma-Aldrich Corporation (St. Louis, MO, USA) unless otherwise indicated.

### Design and preparation of TALENs and CRISPR/Cas9

Both Platinum TALENs and CRISPR/Cas9 (i.e., gRNA) were designed to target the sequence of exon 3 in the pig *SALL1* gene (Fig. 1A). The *SALL1*-targeted Platinum TALEN plasmids were constructed using the Platinum Gate TALEN Kit (Addgene Kit #1000000043) developed by Sakuma *et al*.^53^. The Platinum TALEN plasmids to be used as templates for *in vitro* transcription (IVT) were linearized, and then capped and poly (A)-tailed Platinum TALEN mRNAs were synthesized by using an mMESSAGE mMACHINE T7 Ultra kit (Thermo Fisher Scientific, Waltham, MA) according to the manufacturer’s instructions. For the CRISPR/Cas9 system, four nucleotide spacers (GCGT) and the T7 promoter were added to the gRNA template by polymerase chain reaction (PCR) amplification using the following primers: 5′-GCGTTAATACGACTCACTATAGGGCCGCCCAGGCCGGCAACC and 5′-AAAAGCACCGACTCGGTGCC. The amplified template was gel-purified and used as the template for IVT using a MEGAshortscript T7 kit (Thermo Fisher Scientific). Cas9 mRNA was synthesized by the methods described above using pCAG-hCas9 (Addgene #51142) as a template for IVT. Cas9 protein was obtained from Takara Bio (Shiga, Japan). The Platinum TALEN-encoding mRNAs, gRNA and Cas9 mRNA were purified using a MEGAclear kit and eluted into RNase-free water. These genome-editing molecules were stored at −80 °C until cytoplasmic injection.

### Preparation of pronuclear-stage embryos for cytoplasmic injection

*In vitro* maturation (IVM) of porcine oocytes was performed as described elsewhere^54^. Induction of parthenogenesis and *in vitro* fertilization (IVF) of the IVM oocytes were also performed as reported previously^55^. For parthenogenetic activation, oocytes were washed twice in an activation solution composed of 280 mM mannitol (Nacalai Tesque, Inc., Kyoto, Japan), 0.05 mM CaCl_2_, 0.1 mM MgSO_4_ and 0.01% (w/v) polyvinyl alcohol (PVA). The oocytes were then aligned between two wire electrodes (1.0 mm apart) in a drop of activation solution on a fusion chamber slide (CUY500G1, Nepa Gene, Chiba, Japan). A single direct current pulse of 150 V/mm was applied for 100 µsec using an electrical pulsing machine (LF201; Nepa Gene). Activated oocytes were treated with 5 µg/ml cytochalasin B for 3 h to suppress extrusion of the second polar body.

For IVF, frozen epididymal sperm (1.0 × 10^9^/300 µl) from a WT boar were suspended in 5 ml of Dulbecco’s phosphate-buffered saline (DPBS; Nissui Pharmaceutical Co., Ltd., Tokyo, Japan) supplemented with 0.1% bovine serum albumin (BSA; Wako Pure Chemical Industries, Ltd., Osaka, Japan) and washed three times by centrifugation at 1,000 × g for 4 min. After washing, the precipitated sperm pellet was resuspended in porcine fertilization medium (PFM; Research Institute for the Functional Peptides, Yamagata, Japan) at a concentration of 1 × 10^7^ cells/ml^56^. For insemination, 20 cumulus-oocyte complexes that had been matured *in vitro* were placed in a 100 µl drop of PFM containing spermatozoa (5.0 × 10^4^–1.0 × 10^5^ cells/ml); the oocytes and sperm were incubated for 8 h at 38.5 °C in a humidified atmosphere containing 5% CO_2_, 5% O_2,_ and 90% N_2_. After insemination, the eggs were transferred to HEPES-TL-polyvinylpyrrolidone (PVP); cumulus cells and excess sperm were removed by gentle pipetting. Eggs (i.e., IVM/IVF-derived zygotes) that showed release of polar bodies with normal cytoplasmic morphology were selected for cytoplasmic injection.

Both the parthenogenetic and *in vitro*-fertilized embryos were confirmed in the preliminary experiments to be at the pronuclear stage.

### Cytoplasmic injection

Genome-editing molecules were injected into the cytoplasm of parthenogenetic or IVM/IVF-derived embryos. Cytoplasmic injection was performed by using an inverted microscope equipped with a micromanipulator (MD-102, Narishige, Tokyo, Japan) and an air injector (IG-2, S. Co., Ltd., Tokyo, Japan) at the pronuclear stage, i.e., 4.5–6 h after electrical activation for the parthenotes or 9–10 h after insemination for the IVM/IVF-derived zygotes. Injected embryos were cultured in porcine zygote medium (PZM)-5 medium for 4 days and then cultured until day 7 in PZM-5 supplemented with 10% foetal bovine serum (FBS). After 7 days of culture, the injected embryos were harvested for mutation analysis. For production of foetuses and offspring, injected embryos were transferred at the 1- to 8-cell or blastocyst stage to recipient gilts.

### Embryo transfer

Crossbred (Large White/Landrace × Duroc) prepubertal gilts weighing between 100 and 105 kg were used as recipients for the injected embryos. The gilts were each given a single intramuscular injection of 1,000 IU of equine chorionic gonadotropin (eCG, ASKA Pharmaceutical, Tokyo, Japan) to induce oestrus. Ovulation was induced by an intramuscular injection of 1,500 IU of human chorionic gonadotropin (hCG, Kyoritsu Seiyaku, Tokyo, Japan) 66 h after the injection of eCG. The injected embryos cultured for 1–3 days or 5–6 days were surgically transferred into the oviducts of recipient gilts 52 h (day 1–3 embryos) or 149 h (day 5–6 embryos) after hCG injection under general anaesthesia.

### Analysis of platinum TALEN- and CRISPR/Cas9-induced mutations in blastocysts, foetuses, and piglets

The target regions of *SALL1*-targeted TALENs and CRISPR/Cas9 were amplified by direct PCR from blastocysts using MightyAmp DNA Polymerase Ver.2 (Takara Bio). The primer sequences for Platinum TALENs were 5′-ATTAGGCACCAATGTCGGCAG-3′ and 5′-TGCAGAGCTAAGAGCTGCTCC-3′. The primer sequences for CRISPR/Cas9 were 5′-TGATAGGAAGTCCTCCAACAG-3′ and 5′-AAGAACGCCTCGTCGGAAGC-3′. Nested PCR was then performed using PrimeSTAR GXL DNA Polymerase (Takara Bio), and the appropriate primers were 5′-TGATAGGAAGTCCTCCAACAG-3′ and 5′-TCTCGATGATGACGTTGCTG-3′ for TALENs and 5′-TCGAAGTCACAGGTGGCTCC-3′ and 5′-AAGAACGCCTCGTCGGAAGC-3′ for CRISPR/Cas9. Subsequently, the PCR fragments including the target region were examined using a BigDye Terminator Cycle Sequencing Kit and an ABI PRISM 3130xl Genetic Analyzer (Thermo Fisher Scientific). The sequencing primers were 5′-TCGAAGTCACAGGTGGCTCC-3′ for TALEN and 5′-AGCCAGTCTGCCAGCATCAG-3′ for CRISPR/Cas9.

For analysis of mutations in the foetuses and piglets, genomic DNA was extracted from tail biopsies using a DNeasy Blood and Tissue Kit (Qiagen, Hilden, Germany), and DNA sequencing was then performed as described above.

To determine the incidence of each mutation type in founder SALL1 mutant piglets, genomic DNA was extracted from tail biopsies, gonads (ovaries or epididymal sperm), and kidneys, and the target regions were amplified as described above. PCR products were cloned and sequenced using a Zero Blunt TOPO PCR Cloning Kit for Sequencing (Thermo Fisher Scientific). The 19 to 33 clones for each piglet were analysed.

### Off-target analysis

Off-target sites of *SALL1*-targeted Platinum TALENs in the pig genome were identified by using the online tool PROGNOS (Predicted Report Of Genome-wide Nuclease Off-target Sites; http://bao.rice.edu/Research/BioinformaticTools/prognos.html)^57^. Three of the off-target candidate sites with the highest scores (TALEN scores) were analysed (Supplementary Table S4). Genomic DNA was extracted from the founder foetuses derived from zygotes injected with Platinum TALENs, the region including the off-target candidate sites was amplified by PCR using the appropriate primers, and then the amplicons were sequenced (Supplementary Table S4).

### Immunohistological analysis

Kidney tissues were fixed in a 4% paraformaldehyde solution (FUJIFILM Wako Pure Chemical Corporation, Osaka, Japan), embedded in paraffin, sectioned, and stained with haematoxylin-eosin using standard methods. Immunostaining was carried out using a BlueMap Kit and automated Discovery System (Roche) or manually for immunofluorescence staining.

The following primary antibodies were used: anti-SALL1 (Perseus Proteomics, Tokyo, Japan), anti-WT1 (Santa Cruz Biotechnology), anti-WT1 (Abcam), anti-cytokeratin (Sigma-Aldrich), anti-E-cadherin (BD Sciences), anti-SIX2 (Proteintech, Rosemont, IL), and anti-SALL1 (Abcam), which recognizes the C-terminus of SALL1. Secondary antibodies were conjugated with Alexa 488 or 568 (Thermo Fisher Scientific), and the nuclei were stained with 4′,6-diamidino-2-phenylindole (DAPI). Images were acquired using an LSM780 confocal microscope (Zeiss, Oberkochen, Germany).

### Statistical analysis

Statistical analyses were performed using IBM SPSS Statistics 20.0 software (IBM Corporation, NY, USA). Differences in proportional data between two groups were analysed with the χ^2^ test or Fisher’s exact test. For comparisons among three groups or more, the data were subjected to arcsine transformation and evaluated by one-way analysis of variance (ANOVA) followed by Tukey’s multiple comparisons tests. Differences in blastocyst cell numbers between groups were analysed with ANOVA followed by Tukey’s multiple comparisons tests. The level of significance was set at *P* < 0.05.

## Supplementary information

Supplementary_info

## Data Availability

The datasets generated during and/or analysed during the current study are available from the corresponding author on reasonable request.
