# Supplementary material for: Anephrogenic phenotype induced by SALL1 gene knockout in pigs
Source: Sci Rep. 2019 May 29;9:8016. doi: 10.1038/s41598-019-44387-w (PMC6541644; doi:10.1038/s41598-019-44387-w)
Supplement: Supplementary file 1 — Supplementary_info [file 41598_2019_44387_MOESM1_ESM.docx]

**Supplementary information**

**Anephrogenic phenotype induced by *SALL1* gene knockout in pigs**

Masahito Watanabe^1^, Kazuaki Nakano^2^, Ayuko Uchikura^2^, Hitomi Matsunari^1^, Sayaka Yashima^2^, Kazuhiro Umeyama^1^, Shuko Takayanagi^2^, Tetsushi Sakuma^3^, Takashi Yamamoto^3^, Sumiyo Morita^4^, Takuro Horii^4^, Izuho Hatada^4^, Ryuichi Nishinakamura^5^, Hiromitsu Nakauchi^6,7^, Hiroshi Nagashima^1,2*^

**Supplementary Table S1.** Incidence of mutations in porcine embryos after cytoplasmic injection of CRISPR/Cas9 targeting the *SALL1* gene.

| **Injected molecule** | **Concentration of gRNA/Cas9 (ng/µl)** | **Embryos injected^**^** | **Embryos developed to blastocysts (%)** | **Blastocysts analysed** | **Blastocysts with mutations (%)** |
| --- | --- | --- | --- | --- | --- |
| mRNA | 2/10 | 176 | 87 (49.4) ^a^ | 50 | 31 (62.0) ^a^ |
|  | 5/10 | 177 | 92 (52.0) ^a^ | 54 | 44 (81.5) ^a^ |
|  | 10/10 | 172 | 92 (53.5) ^a^ | 49 | 41 (83.7) ^a^ |
| RNP^*^  (Protein) | 5/2 | 76 | 43 (56.6) ^a^ | 33 | 2 (6.1) ^a^ |
|  | 5/10 | 76 | 48 (63.2) ^a^ | 33 | 21 (63.6) ^b^ |
|  | 5/20 | 82 | 47 (57.3) ^a^ | 33 | 32 (97.0) ^b^ |

^*^ RNP: Ribonucleoprotein complex.

^**^ Parthenogenetic embryos at the pronuclear stage.

^ab^ Values with different superscripts in the same column in each injection experiment differ significantly (P<0.05).

**Supplementary Table S2.** *SALL1* mutations in founder foetuses obtained by genome editing with Platinum TALENs.

| **Foetus** | **Renal formation** | **Target site mutations*** | **Indels** | **Genotype** |
| --- | --- | --- | --- | --- |
| WT | -  - | TCTCCCCCTGAACCCTTCtcccccagtccctctCCCCATCATCCCGATGAA | - | WT |
| M230-1 | Hypoplasia | TCTCCCCCTGAACCCTTCtccccc*****ctctCCCCATCATCCCGATGAA | 5 bp del | 5 bp/13 bp del |
|  |  | TCTCCCCCTGAACCCTTCtccccca*************TCATCCCGATGAA | 13 bp del |  |
| M230-2 | Normal | TCTCCCCCTGAACCCTTCtccccc******tctCCCCATCATCCCGATGAA | 6 bp del | 6 bp/14 bp del |
|  |  | TCTCCCCCTGAACCCTTCtcccc**************ATCATCCCGATGAA | 14 bp del |  |
| M230-3 | Hypoplasia | TCTCCCCCTGAACCCTTCtccc********tctCCCCATCATCCCGATGAA | 8 bp del | 8 bp/14 bp/  969 bp del |
|  |  | TCTCCCCCTGAACCCTTCtcccc**************ATCATCCCGATGAA | 14 bp del |  |
|  |  | TCTCCCCCTGAACCC***********(969 bp)************TTGCC | 969 bp del |  |
| M230-4 | Hypoplasia | TCTCCCCCTGAACCCTTCtccc********tctCCCCATCATCCCGATGAA | 8 bp del | 8 bp/14 bp del |
|  |  | TCTCCCCCTGAACCCTTCtccccc**************TCATCCCGATGAA | 14 bp del |  |
| M230-7 | Hypoplasia | TCTCCCCCCGAACCCTTCtcccc*agtccctctCCCCATCATCCCGATGAA | 1 bp del | 1 bp/2 bp/  2 bp del |
|  |  | TCTCCCCCTGAACCCTTCtcccc**gtccctctCCCCATCATCCCGATGAA | 2 bp del |  |
|  |  | TCTCCCCCTGAACCCTTCtcccT**gtccctctCCCCATCATCCCGATGAA | 2 bp del |  |
| M230-8 | Hypoplasia | TCTCCCCCCGAACCCTTCtcccccagt**C**ccctctCCCCATCATCCCGATGAA | 1 bp ins | 1 bp ins/  13 bp/13 bp/  14 bp del |
|  |  | TCTCCCCCCGAACCCTTCtccccca*************TCATCCCGATGAA | 13 bp del^**^ |  |
|  |  | TCTCCCCCTGAACCCTTCtccccca*************TCATCCCGATGAA | 13 bp del^**^ |  |
|  |  | TCTCCCCCTGAACCCTTCtcccc*a*************TCATCCCGATGAA | 14 bp del |  |
| M230-9 | Hypoplasia | TCTCCCCCTGAACCCTTCtccccca*************TCATCCCGATGAA | 13 bp del | 13 bp del |
| M230-11 | Normal | TCTCCCCCTGAACCCT***************ctCCCCATCATCCCGATGAA | 15 bp del | WT/15 bp del |
| M231-1 | Normal | TCTCCCCCTGAACCCT***************ctCCCCATCATCCCGATGAA | 15 bp del | WT/15 bp del |
| M231-2 | Hypoplasia | TCTCCCCCTGAACCCTTCtccccca*************TCATCCCGATGAA | 13 bp del | 13 bp/  14 bp del |
|  |  | TCTCCCCCTGAACCC**************ctctCCCCATCATCCCGATGAA | 14 bp del |  |
| M231-6 | Hypoplasia | TCTCCCCCTGAACCCTTCtccccca*tccctctCCCCATCATCCCGATGAA | 1 bp del | 1 bp/5 bp del |
|  |  | TCTCCCCCTGAACCCTTCtccccc*****ctctCCCCATCATCCCGATGAA | 5 bp del |  |
| M231-7 | Hypoplasia | TCTCCCCCTGAACCCTTCtccccc********(95 bp)*******GGACA | 95 bp del | 95 bp del |
| M231-8 | Normal | TCTCCCCCTGAACCCTTCtccccca*************TCATCCCGATGAA | 13 bp del | WT/13 bp del |

^*^ Deletions are indicated by *asterisks*. Bold text indicates insertions.

^**^ Two types of 13 bp deletions were detected.

**Supplementary Table S3.** *SALL1* mutations in founder foetuses obtained by genome editing with CRISPR/Cas9.

| **Foetus** | **Renal formation** | **Target site mutations^**^** | **Indels** | **Genotype** |
| --- | --- | --- | --- | --- |
| WT^*^ | - | ggcttcgagcgcgggGGCCGCCCAGGCCGGCAACCCGGccgtctcagcag | - | WT |
| #1705-1 | Normal | ggcttcgagcgcgggGGCCGCCCAGGCCGGCA*CCCGGccgtctcagcag | 1 bp del | 1 bp del/WT |
| #1705-2 | Normal | ggcttcgagcgcgggGGCCGCCCAGGCCGGCA*****************g | 17 bp del | 17 bp del/WT |
| #1705-5 | Hypoplasia | gtgcc***************(1007 bp)*****************gagtc | 1007 bp del | 1007 bp/28 bp/  12 bp del/WT |
|  |  | ggcttcgagc***********(28 bp)***********ccgtctcagcag | 28 bp del |  |
|  |  | ggcttcgagcgcgggGGCCGCCCAGG************ccgtctcagcag | 12 bp del |  |
| #1705-6 | Normal | ggcttcgagcgcgggGGCCGCCCAGGCCGGCAA**A**CCCGGccgtctcagcag | 1 bp ins | 1 bp ins/12 bp del |
|  |  | ggcttcgagcgcgggGGCCGCCCAGGCCG************tctcagcag | 12 bp del |  |
| #1705-9 | Normal | ggcttcgagcgcgggGGCCGCCCAGGCCG************tctcagcag | 12 bp del | 12 bp del/2 bp sub |
|  |  | ggcttcgagcgcgggGGCCGCCCAGGCCGCCCACCCGGccgtctcagcag | 2 bp sub |  |
| #1706-1 | Hypoplasia | ggcttcgagcgcgggGGCCGCCCAGGCCGGC********cgcctcagcag | 8 bp del | 8 bp del |
| #1706-2 | Hypoplasia | ggcttcgagcgcgggGGCC*************ACCCGGccgtctcagcag | 1 bp del | 1 bp/13 bp del |
|  |  | TCTCCCCCTGAACCCTTCtccccc**************TCATCCCGATGAA | 13 bp del |  |

^*^ PAM sequences are underlined.

^**^ Deletions are indicated by *asterisks*. Bold text indicates insertions.

**Supplementary Table S4.** List of off-target candidate sites for the *SALL1*-targeted Platinum TALENs.

| **Off-target candidate site** | **Sequence^*^** | **TALEN score^**^** | **Coordinate** | **Genomic region** |
| --- | --- | --- | --- | --- |
| On-target | 5'-TCTCCCCCTGAACCCTTCtcccccagtccctctCCCCATCATCCCGATGAA-3' | 100 | Chr. 6: 30233117-30233167 | Exon |
|  | 3'-AGAGGGGGACTTGGGAAGagggggtcagggagaGGGGTAGTAGGGCTACTT-5' |  |  |  |
| SALL1-TAL-OTS1 | 5'-TCTCCCCATGGACCCTTCcctggtcctggcctcttaaCCTCCTCTTCCCGATTAA-3' | 65.65 | Chr. 11: 75117568-75117622 | Intergenic |
|  | 3'-AGAGGGGTACCTGGGAAGggaccaggaccggagaattGGAGGAGAAGGGCTAATT-5' |  |  |  |
| SALL1-TAL-OTS2 | 5'-TTCATCGAGGTGATGAGGgacaagacaccttctttgtCTGGCTCATTCCAATGAA-3' | 65.41 | Chr. 15: 21737117-21737171 | Intergenic |
|  | 3'-AAGTAGCTCCACTACTCCctgttctgtggaagaaacaGACCGAGTAAGGTTACTT-5' |  |  |  |
| SALL1-TAL-OTS3 | 5'-TTCATCTGTATGATGGTGagaataactgtacctaccTCATATAATCTCGGTGAA-3' | 65.19 | Chr. X: 57679270-57679323 | Intergenic |
|  | 3'-AAGTAGACATACTACCACtcttattgacatggatggAGTATATTAGAGCCACTT-5' |  |  |  |

^*^ The red and blue letters indicate the left and right TALEN target sequences, respectively. Mismatches are underlined.

^**^ TALEN scores were calculated with the PROGNOS tool (http://baolab.bme.gatech.edu/cgi-bin/prognos/prognos.cgi).

Primer sequences for off-target analysis

| **Off-target candidate site** | **Forward primer** | **Reverse primer** | **Sequence primer** | **Expected PCR product length** |
| --- | --- | --- | --- | --- |
| SALL1-TAL-OTS1 | 5'-CCCCTCCATCACGTTCCACCT-3' | 5'-GAAAGCAGGGAGGTTGATAGCTGG-3' | 5'-TCCACCTTGAGACATCAGC-3' | 421 bp |
| SALL1-TAL-OTS2 | 5'-GCTCCCTGATGGCTTAGGGAGA-3' | 5'-TTTGTCCAGGGTGGGGGTGCT-3' | 5'-GTTATGGAACCAGAGTTCAC-3' | 335 bp |
| SALL1-TAL-OTS3 | 5'-GCCGGGTAGACTCTAGCAGCTT-3' | 5'-GGGGCCATTTCAGCTCTAAGATGC-3' | 5'-GTTCTCCATCTGGAACACAG-3' | 373 bp |

**Supplementary Table S5.** Analysis of mutations in genome-edited founder piglets with mutant *SALL1*.

| **Pig code** | **Sex** | **Target site mutations^*^** | **Indels** | **Genotype** |
| --- | --- | --- | --- | --- |
| WT | - | TCTCCCCCTGAACCCTTCtcccccagtccctctCCCCATCATCCCGATGAA | WT | WT |
| M243-1 | ♀ | TCTCCCCCTGAACCCTTCtc************tCCCCATCATCCCGATGAA | 12 bp del | WT/12 bp/14 bp del |
|  |  | TCTCCCCCTGAACCCTTCtc**************CCCATCATCCCGATGAA | 14 bp del |  |
| M243-2 | ♂ | TCTCCCCCTGAACCCT***************ctCCCCATCATCCCGATGAA | 15 bp del | WT/15 bp del |
| M243-3^#^ | ♀ | TCTCCCCCTGAACCCTTCtc************tCCCCATCATCCCGATGAA | 12 bp del | WT/12 bp del |
| M244-1 | ♀ | TCTCCCCCTGAACCCTTCtccccca*************TCATCCCGATGAA | 13 bp del | WT/13 bp/  660 bp del |
|  |  | AATGGTGGCC**********(660 bp)***********CCGGTGGCTAACA | 660 bp del |  |
| M244-3 | ♂ | TCTCCCCCTGAACCCTTCtccccc*****ctctCCCCATCATCCCGATGAA | 5 bp del | WT/5 bp del |
| M244-4 | ♂ | TCTCCCCCTGAACCCTTCtcccc**************ATCATCCCGATGAA | 14 bp del | WT/14 bp del |
| M244-5 | ♂ | TCTCCCCCTGAACCCTTCtcccc**************ATCCTCCCGATGAA | 14 bp del | WT/14 bp/15 bp/  15 bp del |
|  |  | TCTCCCCCCGAACCCT***************ctCCCCATCATCCCGATGAA | 15 bp del |  |
|  |  | TCTCCCCCTGAACCCT***************ctCCCCATCATCCCGATGAA | 15 bp del |  |
| M244-7 | ♂ | TCTCCCCCTGAACCCTTCtcccccag*ccctctCCCCATCATCCCGATGAA | 1 bp del | WT/1 bp/2 bp del/  19 bp ins |
|  |  | TCTCCCCCTGAACCCTTCtccccca**ccctctCCCCATCATCCCGATGAA | 2 bp del |  |
|  |  | TCTCCCCCTGAACCCTTCtcccccag**GGTTCTCCCTCTCCCTTCTC***ccctctCCCCATCATCCCGATGAA | 19 bp ins |  |
| M253-2 | ♀ | TCTCCCCCTGAACCCTTCtccccc**tccctctCCCCATCATCCCGATGAA | 2 bp del | WT/2 bp/3 bp/  14 bp del |
|  |  | TCTCCCCCTGAACCCTTCtccccc**t*cctctCCCCATCATCCCGATGAA | 3 bp del |  |
|  |  | TCTCCCCCTGAACCCTTCtcccc**************ATCATCCCGATGAA | 14 bp del |  |
| M253-3 | ♀ | TCTCCCCCTGAACCCTTCtccc**a*tccctctCCCCATCATCCCGATGAA | 3 bp del | 3 bp/305 bp del |
|  |  | CCATAGTT*******(305 bp)********tctCCCCATCATCCCGATGAA | 305 bp del |  |
| M253-4 | ♂ | TCTCCCCCTGAACCCTTCtccccc*****ctctCCCCATCATCCCGATGAA | 5 bp del | WT/5 bp del/1 bp/  56 bp ins |
|  |  | TCTCCCCCTGAACCCTTCtcccccag**A**tccctctCCCCATCATCCCGATGAAA | 1 bp ins |  |
|  |  | TCTCCCCCTGAACCCTTCtccc*******AATAAGCAGTTTATTGAGTCCTGCAGCAGCAGCTCCTGCACAGGTACCTCAGCGATCACAA**ccctctCCCCATCATCCCGATGAA | 56 bp ins |  |
| M253-5^**^ | ♂ | TCTCCCCCTGAACCCTTCtccc********tctCCCCATCATCCCGATGAA | 8 bp del | 8 bp/8 bp/15 bp/  15 bp del^***^ |
|  |  | TCTCCCCCTGAACCCTTCtcccc***A*********tCCCCATCATCCCGATGAA | 8 bp del |  |
|  |  | TCTCCCCCTGAACCCT***************ctCCCCATCATCCCGATGAA | 15 bp del |  |
|  |  | TCTCCCCCTGAACCCT***************ctCCCCATCATCCCGATGAA | 15 bp del^***^ |  |
| M253-6 | ♀ | TCTCCCCCTGAACCCTTCtcccc***********A**CCCCATCATCCCGATGAAC | 91 bp ins | WT/91 bp ins |
|  |  | AAATGAATGACACGGTTAACAAAACAGAGCAAGGAGACTGCAGCGACCTTCC |  |  |
|  |  | AGAACACCACGGGCCGGACAGGGAAGAGTCCATGGAGGTGGAGGCCCCGGTG |  |  |
|  |  | GCTAACAAAGGCGGCAGTGGCCC**TTCCAGAACACTGCAGCGACCTTCCAGAA** |  |  |
|  |  | **CACCACGGGCCGGACAGGGAAGAGTCCATGGAGGTGGAGGCCCCGGTGGCTA** |  |  |
|  |  | **ACAAAGGCGGCAGTGGCCC**CCTGGGTGG |  |  |

^#^ Stillborn.

^*^ Deletions are indicated by *asterisks*. Bold text indicates insertions.

^**^ Two types of 8 bp deletions were detected.

^***^ A 487 bp deletion was detected in the second intron of the *SALL1* gene.

**Supplementary Table S6.** Incidence of mutations in various organs of founder piglets with mutant *SALL1*.

| **Pig code** | **Sex** | **Mutation** | **Tail** | **Ovary/sperm** | **Kidney** |
| --- | --- | --- | --- | --- | --- |
| M243-1 | ♀ | 12 bp del | 6.2% (2/32)^*^ | 8.3% (2/24) | 3.6% (1/28) |
|  |  | 14 bp del | 6.2% (2/32) | 8.3% (2/24) | 0% (0/28) |
|  |  | WT | 87.5% (28/32) | 83.3% (20/24) | 96.4% (27/28) |
| M243-2 | ♂ | 15 bp del | 52.6% (10/19) | 45.5% (10/22) | 45.8% (11/24) |
|  |  | WT | 47.4% (9/19) | 54.5% (12/22) | 54.2% (13/24) |
| M244-3 | ♂ | 5 bp del | 36.8% (7/19) | 35.7% (10/28) | 12.0% (3/25) |
|  |  | WT | 63.2% (12/19) | 64.3% (18/28) | 88.0% (22/25) |
| M244-5 | ♂ | 14 bp del | 30.0% (6/20) | 33.3% (8/24) | 16.7% (4/24) |
|  |  | 15 bp del | 10.0% (2/20) | 0% (0/24) | 0% (0/24) |
|  |  | 15 bp del | 15.0% (3/20) | 16.7% (4/24) | 37.5% (9/24) |
|  |  | WT | 45.0% (9/20) | 50.0% (12/24) | 45.8% (11/24) |
| M244-7 | ♂ | 1 bp del | 3.0% (1/33) | 3.7% (1/27) | 0% (0/23) |
|  |  | 2 bp del | 9.1% (3/33) | 0% (0/27) | 0% (0/23) |
|  |  | 19 bp ins | 15.2% (5/33) | 18.5% (5/27) | 8.7% (2/23) |
|  |  | WT | 72.7% (24/33) | 77.8% (21/27) | 91.3% (21/23) |

^*^ The numbers in parentheses indicate the clones analysed.

**Supplementary Table S7.** Nephrogenesis in F1 foetuses of *SALL1*-mutant founder pigs.

| **Generation** | **Pig code** | **Sex** | **Renal phenotype** | **Target site mutations^*^** | **Indels** | **Genotype** |
| --- | --- | --- | --- | --- | --- | --- |
|  | WT | - | - | TCTCCCCCTGAACCCTTCtcccccagtccctctCCCCATCATCCCGATGAA | WT | WT |
| Founder *SALL1* mutant mated pigs | M253-4 | ♂ | NA | TCTCCCCCTGAACCCTTCtccccc*****ctctCCCCATCATCCCGATGAA | 5 bp del | WT/5 bp/  1 bp/56 bp ins |
|  |  |  |  | TCTCCCCCTGAACCCTTCtcccccag**A**tccctctCCCCATCATCCCGATGAA | 1 bp ins |  |
|  |  |  |  | TCTCCCCCTGAACCCTTCtccc*******AATAAGCAGTTTATTGAGTCCTGCAGCAGCAGCTCCTGCACAGGTACCTCAGCGATCACAA**ccctctCCCCATCATCCCGATGAA | 56 bp ins |  |
|  | M244-1 | ♀ | Normal^**^ | TCTCCCCCTGAACCCTTCtccccca*************TCATCCCGATGAA | 13 bp del | WT/13 bp/660 bp del |
|  |  |  |  | AATGGTGGCC**********(660 bp)***********CCGGTGGCTAACA | 660 bp del |  |
| F1 foetuses | W307-1 | ♂ | Normal |  |  | 1 bp ins/WT |
|  | W307-2 | ♀ | Normal |  |  | 5 bp del/WT |
|  | W307-3 | ♀ | Normal |  |  | WT |
|  | W307-4 | ♀ | Normal |  |  | WT/13 bp del |
|  | W307-5 | ♂ | Normal |  |  | 5 bp del/WT |
|  | W307-6 | ♀ | Hypoplasia |  |  | 5 bp/660 bp del |
|  | W307-7 | ♂ | Hypoplasia |  |  | 1 bp ins/660 bp del |
|  | W307-8 | ♂ | Severe Hypoplasia |  |  | 1 bp ins/13 bp del |
|  | W307-9 | ♀ | Hypoplasia |  |  | 1 bp ins/660 bp del |
|  | W307-10 | ♂ | Normal |  |  | WT/660 bp del |
|  | W307-11 | ♂ | Hypoplasia |  |  | 1 bp ins/660 bp del |
|  | W307-12 | ♂ | Normal |  |  | WT/13 bp del |

* Deletions are indicated by *asterisks*. Bold text indicates insertions.

** Normal kidney formation was confirmed at autopsy.

NA: not analysed.


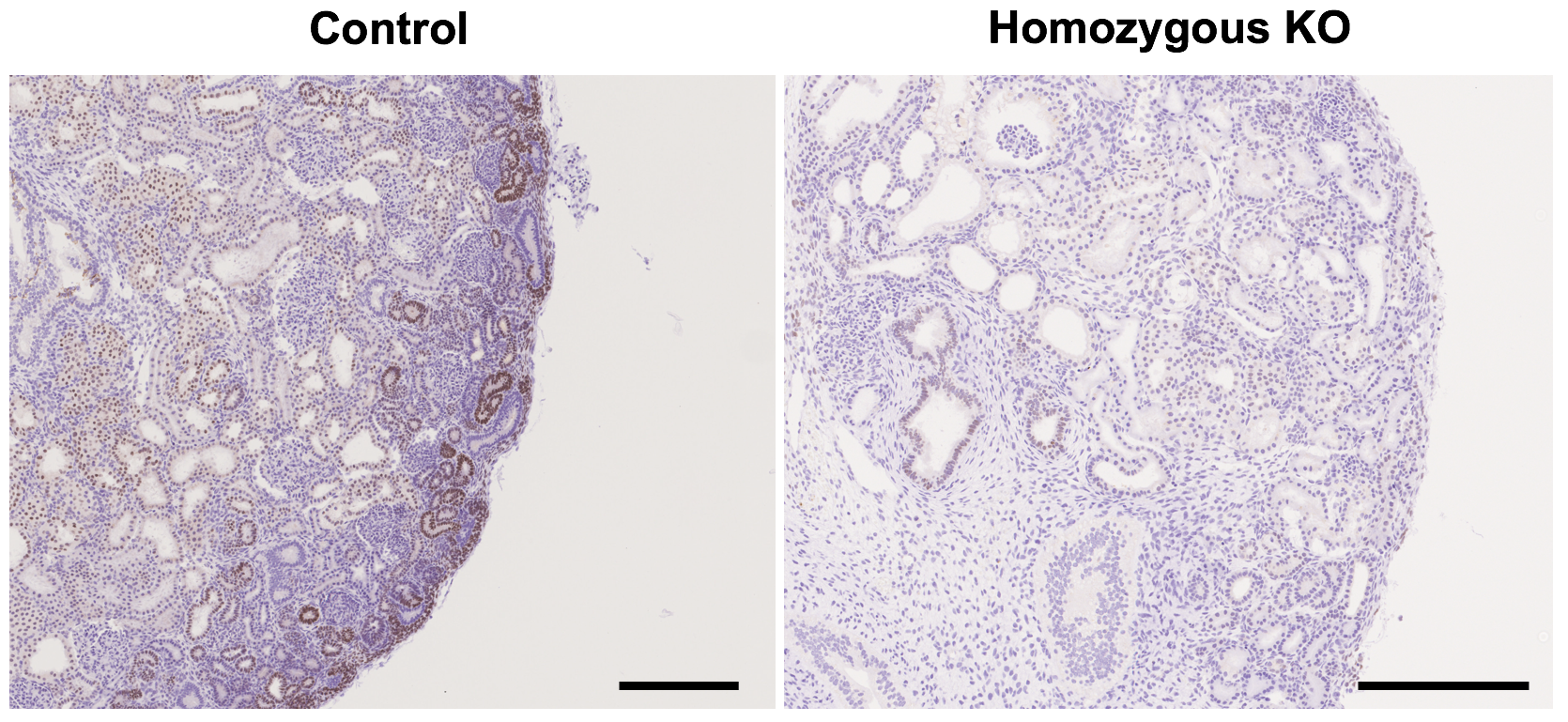


**Supplementary Fig. S1.** **Immunohistochemical analysis of hypoplastic kidneys of F1 progeny carrying large deletion mutations in *SALL1.***

Immunohistochemical staining was performed using an anti-SALL1 antibody that recognizes the C-terminus of the porcine SALL1 protein. F1 foetuses with a homozygous mutation including a 660 bp deletion were produced by mating founder pigs carrying *SALL1* mutations and were obtained on day 40 of gestation. The left and right panels, respectively, show kidney tissue of control (W307-4; WT/13 bp del) and homozygous *SALL1*-KO F1 progeny (W307-7; 1 bp ins/660 bp del). SALL1 expression was not detected in the kidneys of *SALL1*-KO foetuses with the 660 bp deletion. Scale bars: 250 µm.
